# Regional associations of white matter integrity and neurological, post-traumatic stress disorder and autonomic symptoms in Veterans with and without history of loss of consciousness in mild TBI

**DOI:** 10.3389/fnimg.2023.1265001

**Published:** 2024-01-10

**Authors:** Abigail B. Waters, Sarah A. Bottari, Laura C. Jones, Damon G. Lamb, Gregory F. Lewis, John B. Williamson

**Affiliations:** ^1^Brain Rehabilitation Research Center, North Florida/South Georgia VAMC, Gainesville, FL, United States; ^2^Department of Clinical and Health Psychology, University of Florida, Gainesville, FL, United States; ^3^Department of Psychiatry, Center for OCD and Anxiety Related Disorders, University of Florida, Gainesville, FL, United States; ^4^Socioneural Physiology Lab, Kinsey Institute, Indiana University, Bloomington, IN, United States

**Keywords:** TBI, PTSD, MRI, DTI, autonomic, baroreceptor, stress, limbic

## Abstract

**Background:**

Posttraumatic stress disorder (PTSD) and mild traumatic brain injury (mTBI) share overlapping symptom presentations and are highly comorbid conditions among Veteran populations. Despite elevated presentations of PTSD after mTBI, mechanisms linking the two are unclear, although both have been associated with alterations in white matter and disruptions in autonomic regulation. The present study aimed to determine if there is regional variability in white matter correlates of symptom severity and autonomic functioning in a mixed sample of Veterans with and without PTSD and/or mTBI (*N* = 77).

**Methods:**

Diffusion-weighted images were processed to extract fractional anisotropy (FA) values for major white matter structures. The PTSD Checklist-Military version (PCL-M) and Neurobehavioral Symptom Inventory (NSI) were used to determine symptom domains within PTSD and mTBI. Autonomic function was assessed using continuous blood pressure and respiratory sinus arrythmia during a static, standing angle positional test. Mixed-effect models were used to assess the regional specificity of associations between symptom severity and white matter, with FA, global symptom severity (score), and white matter tract (tract) as predictors. Additional interaction terms of symptom domain (i.e., NSI and PCL-M subscales) and loss of consciousness (LoC) were added to evaluate potential moderating effects. A parallel analysis was conducted to explore concordance with autonomic functioning.

**Results:**

Results from the two-way Score × Tract interaction suggested that global symptom severity was associated with FA in the cingulum angular bundle (positive) and uncinate fasciculus (negative) only, without variability by symptom domain. We also found regional specificity in the relationship between FA and autonomic function, such that FA was positively associated with autonomic function in all tracts except the cingulum angular bundle. History of LoC moderated the association for both global symptom severity and autonomic function.

**Conclusions:**

Our findings are consistent with previous literature suggesting that there is significant overlap in the symptom presentation in TBI and PTSD, and white matter variability associated with LoC in mTBI may be associated with increased PTSD-spectra symptoms. Further research on treatment response in patients with both mTBI history and PTSD incorporating imaging and autonomic assessment may be valuable in understanding the role of brain injury in treatment outcomes and inform treatment design.

## 1 Introduction

Posttraumatic stress disorder (PTSD) and traumatic brain injury (TBI) are heterogenous disorders with overlapping symptoms that are overrepresented in active-duty military and Veteran populations. According to the 2008 Rand Report, comorbid PTSD and TBI were seen at a rate of 7%−76% in military members returning from Iraq and Afghanistan (Tanielian et al., 2008), and those with a TBI were three times more likely to be diagnosed with PTSD (Carlson et al., 2011). These conditions are associated with similar symptoms such as memory impairment (Dolan et al., 2012), sleep disturbance/fatigue (Gilbert et al., 2015), emotional lability (O'Neil et al., 2017), depressed mood (Isaac et al., 2015), increased substance use (Miles et al., 2015), and suicidal ideation (Bahraini et al., 2013). Additionally, the triad of depression, history of military mild TBI (mTBI), and PTSD is associated with greater functional disability and unemployment (Lippa et al., 2015; Amick et al., 2018). There are pathophysiological similarities in mTBI and PTSD in that they are both linked to neuroinflammatory, excitotoxic, and oxidative processes that are associated with white matter changes in the brain (Kaplan et al., 2018). With such symptom convergence, similar pathophysiology, and high co-occurrence, it is challenging to disentangle effects of TBI and PTSD and further whether and how TBI contributes to the expression of PTSD. Mechanisms underlying interactions in these conditions remain unclear.

Factor analyses of self-reported symptoms in TBI and PTSD have underscored the difficulty in dissociating effects of co-morbid conditions. The Neurobehavioral Symptom Inventory (NSI; Cicerone and Kalmar, 1995), a self-report measure of post-concussive symptoms, has been found to assess four underlying symptom constructs (i.e., affective, cognitive, somatosensory, and vestibular), based off best fit models from a sample of National Guard members and Veterans with and without history of mTBI and TBI (all severities), respectively, even when controlling for PTSD (Benge et al., 2009; Vanderploeg et al., 2015). However, correlations between post-concussive symptoms and PTSD symptoms (i.e., re-experiencing, avoidance, hyperarousal, and negative alterations in cognition or mood) are high, possibly due to both overlapping pathophysiology and item characteristics as −15% of items have direct crosswalks to items on common PTSD self-report measures (O'Neil et al., 2021; Scimeca et al., 2021). Furthermore, military service members diagnosed with both PTSD and TBI tend to have greater PTSD symptom severity and higher rates of disability in comparison to those with PTSD only (Lippa et al., 2015). The overlap in symptom presentation and heightened severity of PTSD is possibly due, in part, to commonly affected brain networks in TBI that may contribute to similar symptoms to PTSD as well to increased expression of symptoms of PTSD.

In mild-to-moderate TBI, the observed variability in white matter is likely due to some combination of both premorbid vulnerability and secondary injuries, as direct, primary injuries (e.g., hemorrhage) are relatively less common (Blennow et al., 2012). However, diffuse axonal injury (DAI) via shearing injury is still possible in this population. Notably, frontolimbic pathways responsible for executive functioning and emotion regulation are some of the most vulnerable areas for damage (Wright et al., 2017; Badea et al., 2018; Kulkarni et al., 2019). Following injury, a multi-faceted neurometabolic cascade (i.e., secondary injuries) occurs in which there is a depolarization of the neuron followed by dysregulated glutamate release, thereby triggering a surge in energy demand and a temporary metabolic crisis, contributing to further axonal damage (Giza and Hovda, 2014); this is detectable in blood, even in mTBI (Wang et al., 2021). This is the proposed substrate for acute post-mTBI symptoms with other factors such as age, history of prior concussion, and comorbid neurological and psychiatric conditions moderating recovery (Bonfield et al., 2013; Karr et al., 2014).

While PTSD is thought to be a predominately psychiatric condition, there have been reports of white matter alteration in PTSD, although the mechanisms for these changes are not fully understood. In recently deployed military service members, reduced fractional anisotropy (FA; the most common diffusion-based indicator of underlying white matter structure) of frontolimbic white matter tracts (e.g., the uncinate fasciculus) was associated with startle and subthreshold PTSD symptomology (Costanzo et al., 2016). Further, in a study comparing civilians and Veterans with mTBI, Davenport et al. (2016) found that environment in which mTBI occurred was not determinant of weaker FA connections but that lifetime history of PTSD significantly interacted with deployment mTBI. Contrasting these findings, numerous diffusion-weighted imaging (DWI) studies of people with comorbid PTSD and TBI found no or little difference among common DWI metrics, Bazarian et al. (2013); Petrie et al. (2014); Lopez et al. (2017); Yeh et al. (2017); Bolzenius et al. (2018), and Santhanam et al. (2019), inconsistent significant regions of interest (ROI), and little evidence tying an association between DWI measures and PTSD with either small effect size or low predictive variance (Bazarian et al., 2013; Lepage et al., 2018). However, it is widely acknowledged in the literature that the high cooccurrence of PTSD and mTBI can exacerbate one another both neurophysiologically and symptomatically, but the mechanisms are not yet fully understood (Daniels et al., 2013; Davenport et al., 2016; O'Doherty et al., 2018; Santhanam et al., 2019).

Studies investigating the neurobehavioral outcomes following mTBI have consistently found that the presence of comorbid PTSD is linked to poorer results on a range of assessments, including self-reports of emotional regulation and global functioning (e.g., social engagement, disability) (Pietrzak et al., 2009; Macera et al., 2012; MacDonald et al., 2014; Combs et al., 2015; Haagsma et al., 2015; Jackson et al., 2016). Following TBI, dysfunction within fronto-limbic pathways may impact the ability to inhibit down-up neurophysiological reactivity (i.e., fight-flight, behavioral immobilization) expressed in PTSD (Williamson et al., 2013). This failure of inhibition may lead to disruptions in autonomic systems (Shah et al., 2013), increasing the effects of chronic stress on overall health and daily functioning. Autonomic dysregulation is a core feature of PTSD, criterion D. Hyperarousal symptoms are characterized by increased restlessness, startle response/stressor reactivity, and decreased sleep quality. Chronic hyperarousal is associated with accelerated physiological aging as evidenced by increased DNA methylation (Wolf et al., 2016). While criterion D is typically assessed with self-report, objective autonomic measures are different in patients with PTSD. High frequency heart rate variability, an index of vagal contributions to interbeat variability of the heart, is lower in patients with PTSD (Schneider and Schwerdtfeger, 2020), and, furthermore, responsive to successful treatment with psychotherapy (Shah et al., 2013). High frequency heart rate variability is associated with a variety of health outcomes; lower heart rate variability is associated with metabolic risk (Wulsin et al., 2015), pulmonary disease (Alqahtani et al., 2023), cardiovascular disease, and all-cause mortality (Jarczok et al., 2022). Parasympathetic control is also a critical system in inhibiting sympathetic nervous system response to stress, aka the vagal brake (Porges, 2001; Thayer and Lane, 2009). Frontally mediated networks of critical in autonomic control (Beissner et al., 2013) and disruption in key fronto-limbic white matter pathways may bias autonomic mobilization toward a maladaptive response to stressors; i.e., disrupting this system may be a critical factor in the amplification of PTSD symptoms associated with TBI. Understanding the relationship between symptom severity, autonomic functioning, and symptoms domains in fronto-limbic pathways will help to identify potential treatment targets when dissociating the effects of potentially interacting conditions.

The primary aim of this study was to determine if there was regional variability in the relationship between white matter integrity in major tracts and symptom severity in a mixed sample of Veterans with PTSD and/or mTBI (i.e., are some white matter tracts more closely associated with symptom severity in PTSD and mTBI compared to other white matter tracts?). We assessed symptom severity as a global construct, as well as the potential moderating effect of symptom domain (e.g., trauma-related re-experiencing, post-concussive vestibular complaints) and initial injury severity [i.e., loss of consciousness (LoC) following TBI]. A secondary aim of this study was to contrast analyses of symptom severity with objective measures of autonomic functioning [i.e., respiratory sinus arrythmia (RSA) and baroreceptor sensitivity (BRS)], in order to explore potentially overlapping pathophysiological mechanisms in PTSD and TBI. Examining the discriminant associations between tracts, autonomic functioning, and symptom severity in PTSD and TBI will provide a better understanding of the relationship between these two, often overlapping, conditions.

## 2 Methods

### 2.1 Participants

One-hundred and forty Veterans were screened for participation. Ninety-four Veterans who were previously deployed on active duty to a theater of combat operations were recruited from the North Florida/South Georgia Department of Veterans Affairs Medical Center and surrounding community. Eleven participants were excluded after enrollment and 6 individuals did not complete the MRI; 77 Veterans were included in the final analysis. Our sample consisted of 70 men and 7 women, age ranged from 23 to 45 years in age (*M* = 32.14 years, *SD* = 6.38), with an average of 14.34 years of education (*SD* = 1.95; see Table 1).

**Table 1:** Two group demographic table.

	**Overall Sample (n = 77)**	**Hx of mTBI with LOC (*N =* 34)**	**No Hx of mTBI with LOC (*N =* 43)**	**Group comparison**
Age (M±SD)	32.14 ± 6.38	33.50 ± 6.07	31.00 ± 6.48	W = 539.50, *p < * 0.05^*^ (Wilcoxon rank sum test)
Sex (% Male)	90.9%	91.2%	90.7%	*p =* 1.00 (Fisher's Exact Test)
Education (M±SD)	14.34 ± 1.95	14.4 ± 2.13	14.3 ± 1.82	W = 749.00, *p =* 0.85 (Wilcoxon rank sum test)
Race (%)	67.5% White 18.2% Black 14.3% Other	64.7% White 23.5% Black 11.8% Other	69.8% White 13.9% Black 16.3% Other	*p =* 1.00 (Fisher's Exact Test)
Number of TBIs (M ± SD)	1.68 ± 1.64	2.35 ± 1.87	1.14 ± 1.21	W = 423.50, *p < * 0.01^**^ (Wilcoxon rank sum test)
PTSD diagnosis (%)	48.1%	64.7%	34.9%	*x*^2^(1) = 5.62, *p =* 0.018^*^
BDI-II total	15.71 ± 12.45	21.60 ± 12.30	11.10 ± 10.60	W =354.00, *p < * 0.01^***^ (Wilcoxon rank sum test)

Individuals without a history of mTBI with LOC may have reported a mTBI with only alterations of consciousness or no mTBI. LOC, Loss of Consciousness; Other, multi-racial or no response; TBI, Traumatic Brain Injury; BDI-II, Beck Depression Inventory-II; ^*^p < 0.05, ^**^p < 0.01, ^***^ p < 0.001.

Veterans included those with and without history of TBI, with and without history of PTSD, and neither. mTBI diagnosis was determined according to VA/DOD diagnostic guidelines using the Ohio State University TBI Identification Method- Short Form (Corrigan and Bogner, 2007). Participants were also assessed for PTSD symptoms and coded as having a PTSD diagnosis if the Veteran (1) reported experience of a Criterion A traumatic event on a structured clinical interview designed for this study, and (2) endorsed current symptoms meeting DSM-IV diagnostic criteria on the PTSD Checklist- Military version (PCL-M; Weathers et al., 1993). Participants' self-report was verified via review of the VA Computerized Patient Record System (CPRS) and military service medical records, when available. A diagnostic consensus conference with a licensed clinical psychologist and neuroscientist was used to verify each participant's diagnostic group.

Moderator analyses were completed to examine the impact of LoC in mTBI, as a proxy variable for injury severity. All individuals in the “LoC” group were by definition mTBI subjects. The “no LoC” group included a mix of participants with mTBI (but without LoC) and without any reported mTBI. This grouping variable was chosen for two reasons. First, although individuals with and without LOC meet VA/DOD diagnostic guidelines for mTBI, duration of LoC is a commonly accepted marker of injury severity. Using LoC as a categorical grouping allowed us to probe the heterogeneity within mTBI. Due to sample size, we were unable to fully parse the differences between mTBI with and without LoC. Second, in this population of Veterans with combat exposure and mTBI and/or PTSD, retrospective reports of alterations of consciousness (e.g., feeling “dazed”) can be easily conflated with acute stress responses. The presence of LoC reflects a “confirmed” mTBI, as opposed to ambiguous or absent mTBI in the “no LoC” group. Exclusion criteria were: neurological disorders other than TBI, major medical conditions, severe psychiatric conditions other than PTSD and likely unrelated to trauma (e.g., schizophrenia), premorbid (to trauma) sleep disorders, self-report of current substance abuse (within the past 2 weeks for marijuana or alcohol and within the past 2 months for other substances), current prescription for medications that influence autonomic activity (e.g., beta blockers, angiotensin-converting enzyme inhibitors), pregnancy, and any contraindications to MRI scanning. All study procedures were approved by the University of Florida Institutional Review Board. Participants provided written informed consent and were compensated for their time and travel.

Note, these data analyzed in the present manuscript are from an existing dataset that includes multimodal neuroimaging, autonomic and neurobehavioral metrics. There are prior publications with portions of this sample (Lamb et al., 2017; Bottari et al., 2021; Rieke et al., 2021).

### 2.2 Measures

#### 2.2.1 PTSD checklist-military version

Participants self-reported PTSD symptom severity using the PTSD checklist- military version (PCL-m; Weathers et al., 1993). Total symptom severity scores were obtained by summing participants' responses across each of the 17 items. Total symptom severity scores can range from 17 to 85 with higher scores indicating greater PTSD symptom severity. Subscores were also calculated for each DSM-IV PTSD symptom cluster by summing items on the PCL-m relating to re-experiencing (items 1–5), avoidance (items 6–12), and hyperarousal (items 13–17) (Williams et al., 2011).

#### 2.2.2 Neurobehavioral symptom inventory

Participants self-reported on post-concussive symptom severity using the 22-item neurobehavioral symptom inventory (NSI; Cicerone and Kalmar, 1995). Subscores were calculated for each NSI factor (vestibular, somatic, cognitive, and affective) based on the four-factor, 20-item NSI model that achieved the best fit as described in Vanderploeg et al. (2015). This model excludes two items (hearing problems and appetite disturbance) due to poor fit. Higher scores on each subscale are indicative of greater post-concussive symptom severity.

#### 2.2.3 Neuroimaging

All participants underwent scanning with a 3-t Philips achieva MRI. t1-weighted, t2-weighted, 3d fluid attenuated inversion recovery (FLAIR), t2^*^-weighted echo planar, and high angular resolution diffusion imaging (HARDI) sequences were acquired. The diffusion gradients were applied along 6 directions with a *b*-value of 100 s/mm^2^ and along 64 non-collinear directions using a *b*-value of 1000 s/mm^2^. One image was acquired with diffusion weighting (*b* = 0).

Anatomical reconstruction was performed on each participant's T1-weighted image using FreeSurfer software to obtain a cortical parcellation and subcortical segmentation for each participant (Fischl, 2012). Diffusion tractography was performed using FreeSurfer's (v6.0) TRActs Constrained by UnderLying Anatomy (TRACULA) tool, which provides an automated probabilistic reconstruction of white matter pathways from each participant's DWI data (Yendiki et al., 2011). This approach has a number of advantages including (1) the use of a probabilistic ball-and-stick model of tractography allows for the modeling of the white matter tract in areas of high local uncertainty (e.g., crossing fibers, low anisotropy), (2) the reconstruction is completed in each subject's native space to reduce estimation errors and (3) characterizes significant variability over the length of the white matter tract, reducing the likelihood of spurious findings. During pre-processing, data were corrected for head motion-related artifacts and image distortion due to eddy currents using eddy_correct in the FSL (v6.0) toolbox (https://fsl.fmrib.ox.ac.uk/fsl/fslwiki). In addition, data were visually inspected before and after processing and no apparent issues with motion-related artifacts were identified. The DTI parameter FA was calculated using dtifit tools in FSL. The average FA for the center of each major white matter tract was used for statistical analysis to avoid non-white matter tissue partial volume effects (Alexander et al., 2001; Roine et al., 2014). Analyses included inferior longitudinal fasciculus (ILF), uncinate fasciculus (UNC), anterior thalamic radiations (ATR), cingulum-cingulate gyrus (supracallosal) bundle (CCG), cingulum-angular (infracallosal) bundle (CAB), superior longitudinal fasciculus-parietal bundle (SLFP), superior longitudinal fasciculus-temporal bundle (SLFT), forceps major (Fmajor), and forceps minor (Fminor).

#### 2.2.4 Autonomic functioning

Respiratory sinus arrhythmia (RSA) was calculated from data collected during a posturally-modulated (standing to laying face up at an angle) autonomic assessment using a tilt table. Heart variability statistics were derived using the Porges–Bohrer method (Lewis et al., 2012) using customized software (Brain-Body Center, 2007; Brain-Body Center for Psychophysiology and Bioengineering, 2016). Heart rate, recorded with a 3-lead electrocardiogram, and continuous arterial blood pressure were collected for 3 min in each position-−90° (standing/upright), 60°, 30°, 60°, and 90°–with slow transitions in between (~2°s/s). The primary metric used in analysis was the difference in RSA from 30° tilt to the second 90° tilt (returned to upright), as this represents the largest positional shift from supine. A difference score for baroreceptor sensitivity (BRS) was calculated using the same method, for a subset of individuals (*n* = 37).

### 2.3 Statistical analysis

#### 2.3.1 Pre-analysis

Descriptive statistics were used to report participant demographics and scores for symptom scales (i.e., PCL-m and NSI). PCL-m and NSI scores were z-scaled within this sample, with more positive scores representing more severe reported symptoms. The average FA for each tract was z-scaled within tract, with more positive scores representing increased FA.

#### 2.3.2 Mixed-effects modeling

Statistical analysis was conducted in r 4.1.2, modeled on the approach used by Mace et al. (2019). Mixed effects models were conducted with the lme4 package (Bates et al., 2015). Mixed-effects models have a number of advantages over traditional linear regression (Baayen et al., 2008), and are ideal for high-dimensional neuroimaging data with multiple measurements per subject (e.g., FA for each tract). Values from each participant can be imputed with no prior aggregation; thus, both by-item and by-participant variation are accounted for in a single model (Winter, 2013) through both fixed and random effects. Mixed-effects modeling allowed us to simultaneously quantify which tracts were most related to both total symptom severity and specific sub-domains of symptomatology. Principal components analysis (Varimax rotation) of NSI and PCL-m subscales, in addition to prior literature (Hoover et al., 2022), provided support for testing global symptom severity via mixed-effects models (KMO = 0.86; Bartlett's test, *p* < 0.001; standardized factor loadings 0.72–0.93; variance explained 74.0%).

Linear mixed-effects models included symptom subscales on the NSI and PCL-M (Domain; factor, 7 levels), symptom severity scores agnostic to domain (Score; continuous), white matter tract (Tract; factor, 9 levels), and LoC (factor, 2 levels), as predictors of tract FA (continuous criterion). Random intercepts were included for inter-subject and hemispheric variability. Age and sex were entered as covariates of non-interest. Akaike information criterion (AIC) and F-tests of the variance ratio were compared between nested models. The Score × Tract interaction was used to assess the regional specificity of associations between global symptom severity and white matter. The Score × Tract × Domain interaction was used to determine if the Score × Tract relationship varied by symptom subscales, which aids in differentiating between PTSD and TBI pathophysiology. The Score × Tract × LoC interaction examined whether there was a moderating effect of TBI. A planned, parallel analysis was conducted with measures of autonomic function (RSA; continuous), testing the RSA × Tract × LoC interactions. An exploratory analysis with BRS (continuous) was also conducted to parallel the model used with RSA. Predicted marginal means (i.e., slopes) for the relationship between FA and predictors were estimated with the *lsmeans*, with 95% confidence intervals (CI) generated using 1000 bootstrapped samples, in order to more comprehensively quantify the strength and reliability of findings.

## 3 Results

### 3.1 Pre-analysis

In our study of Veterans who had been deployed in combat, we observed a wide spectrum of symptoms related to PTSD and a history of mTBI. We divided the sample into those with and without a history of LoC. Demographic characteristics of our sample were largely representative of the local veteran population, which is predominantly White, male, and has some college education on average. Groups (LoC vs. no LoC) differed on age (*W* = 539.5, *p* = 0.0497), number of TBIs (*W* = 423.5, *p* = 0.0012), alteration of consciousness (AoC) (*p* < 0.001), and PTSD diagnosis (*x*^2^ = 5.62, *p* = 0.018) (see Table 1). Those with history of LoC had an average raw NSI score of 31.9 (*SD* = 20.8) compared to 14.2 (*SD* = 12.6) for those without LoC. History of LoC was positively and strongly associated (*p* < 0.001) with total scores and all domains of the PCL-M and NSI, except for the Cognitive domain of the NSI (*p* < 0.01) (see Supplementary Table 1). On the PCL-M, a similar pattern was seen with those with history of LoC having a higher average raw score (*M* = 49.4, *SD* = 18.8) than those without (*M* = 31.7, *SD* = 13.4). Correlations between symptom subscales were high, ranging from 0.54 to 0.94, with all reaching significance (see Table 2). There were no significant correlations between RSA Difference Score and the PCL-M and NSI (*p* > 0.05) (see Table 2).

**Table 2:** Descriptive statistics and correlations for PCLM and NSI.

	**Descriptive statistics**						**Spearman correlations**								
**Domain**	**M**	**SD**	**Min**	**Max**	**Skew**	**Kurtosis**	**1**	**2**	**3**	**4**	**5**	**6**	**7**	**8**	**9**
1. PCLM total	39.49	18.22	17	84	0.72	−0.48									
2. PCLM avoidance/numbness	15.87	8.16	0	35	0.64	−0.56	0.94								
3. PCLM hyperarousal	14.34	6.82	5	29	0.51	−0.85	0.93	0.85							
4. PCLM reexperiencing	10.75	10.75	0	25	0.67	−0.41	0.90	0.80	0.77						
5. NSI total	22.03	18.85	0	80	1.13	0.56	0.84	0.81	0.88	0.66					
6. NSI affective	7.99	6.52	0	24	0.78	−0.40	0.81	0.82	0.88	0.63	0.94				
7. NSI cognitive	4.96	4.44	0	16	0.89	−0.12	0.75	0.74	0.79	0.54	0.89	0.82			
8. NSI SOMATIC	5.44	5.61	0	28	1.61	2.80	0.75	0.70	0.75	0.66	0.90	0.79	0.72		
9. NSI vestibular	1.83	2.37	0	11	1.63	2.74	0.61	0.59	0.70	0.49	0.80	0.72	0.71	0.71	
10. RSA difference score	0.54	1.04	−3.44	2.36	−1.12	2.57	0.05	0.04	−0.02	0.11	0.03	−0.03	0.01	0.10	−0.06

RSA, Respiratory sinus arrhythmia; NSI, Neurobehavioral Symptom Inventory; PCLM, PTSD Checklist Military. All correlations were significant (*p* < 0.001), except for RSA Difference Score (*p* > 0.05).

### 3.2 Mixed effects modeling

#### 3.2.1 Predictors of global FA

Mixed effects modeling of single predictors (see Table 3) suggested that that there was no relationship between global FA and global symptom severity [score; *F*_(1, 8192)_ = 0.00, *p* = 0.950], injury severity characteristics [LoC; *F*_(1, 71)_ = 2.35, *p* = 0.130], or autonomic functions [BRS, *F*_(1, 33)_ = 2.50, *p* = 0.123; RSA, F_(1, 58)_ = 1.40, *p* = 0.242]. FA also did not significantly vary between tracts [*F*_(8, 1999)_ = 0.00, *p* > 0.999], as would be expected following z-scaling. A *post-hoc* analysis examining the raw FA values demonstrated some variability between tracts, with the CAB having the lowest average FA and the FMajor having the highest (see Supplementary Table 2).

**Table 3:** Results from mixed effects models including main effects and interactions.

**Predictor**	**N_parameters_**	**AIC**	***F***	***X^2^***
1. Tract	14	15112	0.00	—
2. Score	7	15098	0.00	—
3. Domain	12	15108	0.00	—
4. LoC	7	15096	2.35	—
5. Score × Tract	23	15079	6.41^***^	51.29^A***^
6. Score × Tract × Domain	131	15285	0.22	10.42^B^
7. Score × Tract × LoC	41	15022	6.31^***^	93.33^B***^
8. RSA	7	12436	1.40	—
9. RSA × Tract	23	12270	16.06^***^	150.40^C***^
10. RSA × Tract × LoC	41	12237	3.51^***^	69.49^D***^

^*A*^Two-way interactions were compared to model 1. ^*B*^Compared to model 5. ^*C*^Compared to model 8. ^*D*^Compared to model 9. ^***^p < 0.001.

#### 3.2.2 Tract specificity for global symptom burden and autonomic burden

Results from mixed effects modeling of two-way score × tract interaction (see Table 3) suggested that there was regional specificity in the relationship between FA and global symptom severity [*F*_(8, 5071)_ = 6.41, *p* < 0.001]. Bootstrapped estimates of slopes (see Table 4) revealed that the global symptom severity was significantly associated with FA in 2 tracts. For every 1 SD increase in global symptom severity there was a 0.07 SD increase in FA within the CAB (95% CI [0.01, 0.12]) and a 0.09 SD decrease in FA within the UNC (95% CI [−0.05, −0.13]). However, the addition of symptom subscale (score × tract × domain interaction) did not improve overall model fit, suggesting that the effects seen in the UNC and CAB did not vary by symptom domain (TBI vs. PTSD).

**Table 4:** Bootstrapped (*B* = 1,000) slope estimates and 95% confidence intervals (CI) from the two-way Score × Tract and RSA × Tract interactions.

	**Score** × **Tract**			**RSA** × **Tract**		
**Tract**	**Slope**	**Lower CI**	**Upper CI**	**Slope**	**Lower CI**	**Upper CI**
Anterior thalamic radiation	−0.03	−0.07	0.01	**0.28**	**0.25**	**0.30**
Cingulum angular bundle	**0.07**	**0.01**	**0.12**	0.02	−0.04	0.07
Cingulum cingular bundle	0.02	−0.02	0.06	**0.06**	**0.02**	**0.09**
Forceps major	−0.02	−0.07	0.04	**0.09**	**0.05**	**0.13**
Forceps minor	0.04	−0.01	0.10	**0.06**	**0.02**	**0.09**
Inferior longitudinal fasciculus	−0.02	−0.05	0.02	**0.09**	**0.06**	**0.11**
Superior longitudinal fasciculus– parietal endings	0.03	−0.01	0.07	**0.16**	**0.12**	**0.19**
Superior longitudinal fasciculus– temporal endings	0.00	−0.04	0.04	**0.14**	**0.11**	**0.17**
Uncinate fasciculus	**−0.09**	**−0.13**	**−0.05**	**0.19**	**0.16**	**0.22**

Significant levels (p < 0.05) were bolded.

**Figure 1:**
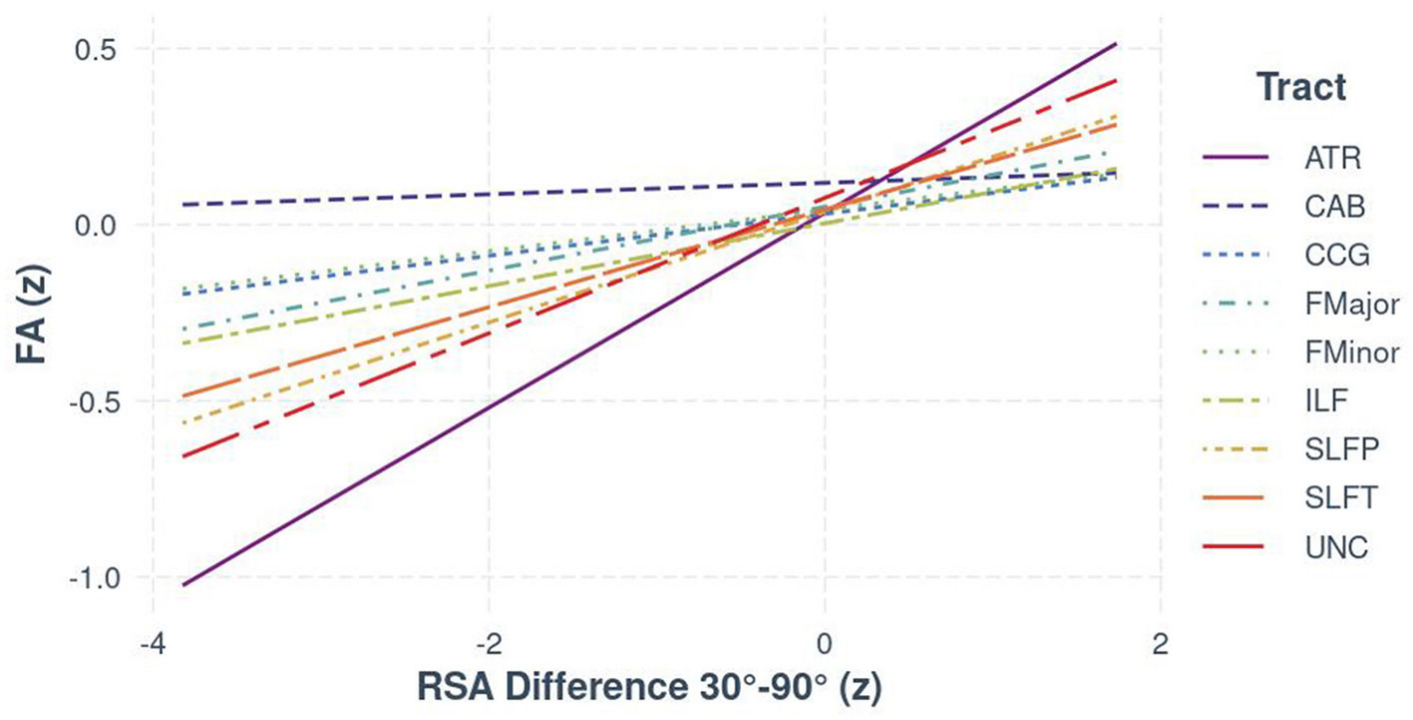
The relationship between FA and RSA (z-standardized scores), separated by white matter tract for the full sample.

Results from the RSA × Tract interaction (see Table 3) also suggested there was regional specificity in the relationship between FA and autonomic function [*F*_(8, 1602)_ = 16.06, *p* < 0.001; see Figure 1]. Bootstrapped estimates of the slopes (see Table 4), revealed that RSA was positively associated with FA in all tracts, except the CAB (95% CI [−0.04, 0.07]). For significant tracts, the estimated slopes ranged from 0.06 to 0.28, with the strongest relationships observed in the ATR (95% CI [0.25, 0.30]) and the UNC (95% CI [0.16, 0.22]). A similar pattern was seen for the exploratory BRS × Tract interaction [*F*_(8, 967)_ = 16.18, *p* < 0.001], despite the reduced sample size, with positive relationships between FA and BRS in all tracts, except the CAB (95% CI [−0.07, 0.08]).

#### 3.2.3 Moderating effect of TBI with LoC

The moderating effect of LoC in TBI was estimated using two, three-way interaction terms in parallel models: Score × Tract × LoC and RSA × Tract × LoC. Results (see Table 3) suggested that LoC moderated the association for both global symptom severity [*F*_(8, 5862)_ = 6.31, *p* < 0.001] and RSA [*F*_(8, 1595)_ = 3.51, *p* < 0.001].

**Figure 2:**
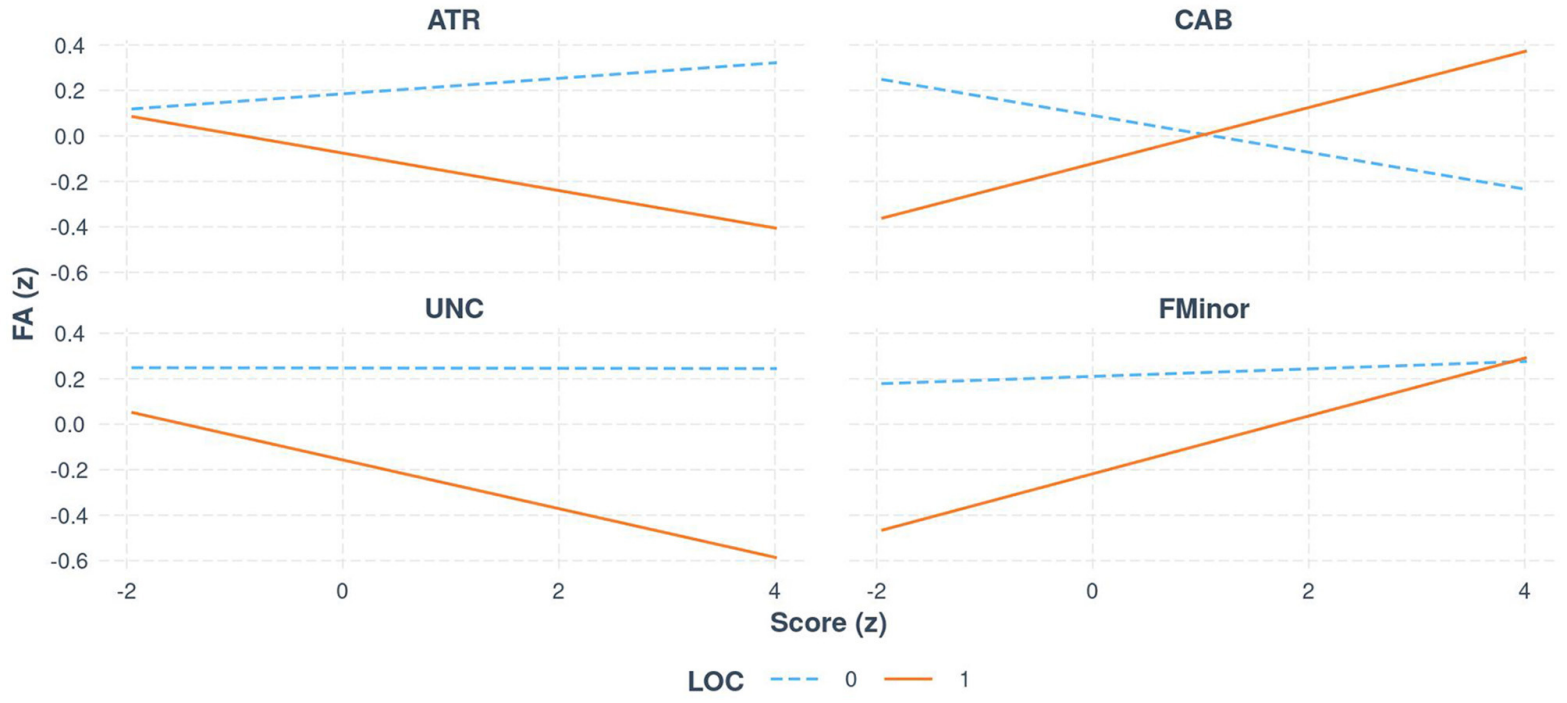
The relationship between FA and global symptom score (z-standardized scores), for select white matter tracts, separated by group (LoC vs. no LoC).

Bootstrapped estimates of slopes suggested that, for individuals without LoC, there was no significant relationship between FA and Score across tracts. For individuals with LoC, there was a significant relationship between FA and global symptom severity in 4 tracts (see Figure 2 [Interaction Line plots]). For every 1 SD increase in global symptom severity, there was a 0.10 and 0.08 decrease in SD of FA of the UNC (95% CI [−0.05, −0.17]) and ATR (95% CI [−0.03, −0.13]) respectively, but a 0.13 increase in both the CAB (95% CI [0.05, 0.20]) and Forceps Minor (95% CI [0.06, 0.19]).

**Figure 3:**
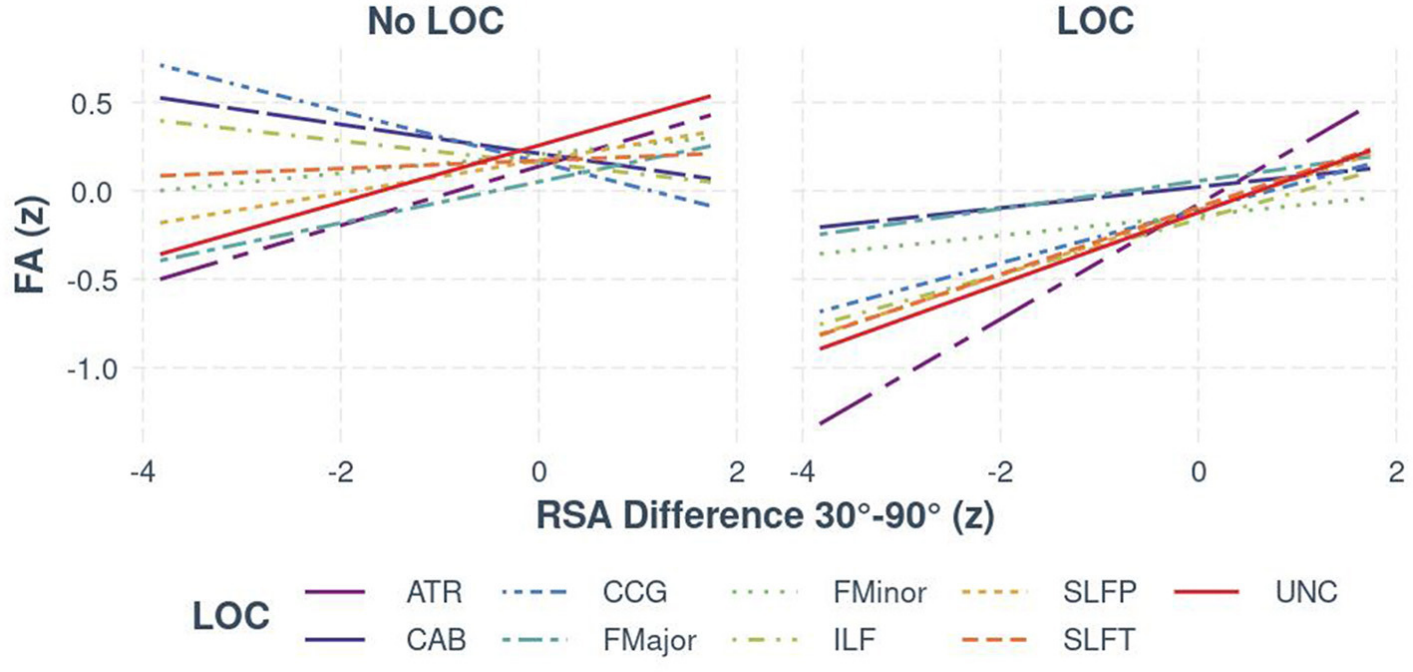
The relationship between FA and RSA (z-standardized scores), separated by white matter tract and group (LoC vs. no LoC).

Regarding autonomic functioning, for individuals with LoC, bootstrapped estimates of slopes mirrored the findings from the two-way interaction; RSA was positively associated with FA in all tracts, with the weakest relationship observed in the CAB (95% CI [0.01, 0.11]) and the strongest relationships in the ATR (95% CI [0.30, 0.35]) and the UNC (95% CI [0.17, 0.23]). For individuals without LoC, there was a weaker, but still significant, positive relationship in the ATR, SLFP, and UNC only, with slopes ranging from 0.02 to 0.16. The relationship between RSA and both interhemispheric pathways (FMajor and FMinor) was not meaningfully different between those with and without LoC. There was a significant negative relationship between FA and RSA in the CCG (95% CI [−0.07, −0.21]) [see Figure 3 (Visualization of bootstrapped CIs, grouped by LoC and no LoC for the RSA × Tract × LoC interaction)]. For the exploratory analysis with BRS, the results remained consistent in the limbic pathways (ATR, UNC, and CCG), despite the reduced sample size.

## 4 Discussion

This study demonstrated regional specificity in the relationship between white matter integrity and global symptom severity, as well as between white matter and autonomic functioning in combat exposed Veterans with and without history of mild TBI and PTSD. These relationships were further moderated by LoC in those with mTBI, suggesting that injury severity during TBI may interact with chronic stress via impact on critical structures in regulating stress (although causality cannot be determined by this study). Consistent with the growing body of literature surrounding symptom typology in PTSD and TBI, our findings did not suggest that symptom domain (i.e., NSI vs. PCL-M) explained additional variance in white matter. These findings may have important implications for the clinical management of PTSD and TBI in Veterans.

Across the full sample, global symptom severity was positively associated with FA in the CAB and negatively associated with FA in the UNC. It is possible that the positive association between symptom severity and FA in the CAB reflects the loss of a sub-section crossing fibers (Bazarian et al., 2007), resulting in a paradoxical increase in FA as white matter degrades. Within the ventro-limbic system of the brain, the CAB and UNC are two pathways implicated in emotional regulation, memory, and autonomic feedback, that may play a role in the maintenance of symptoms (Williamson et al., 2013; Averill et al., 2018; Bottari et al., 2021). However, it is important to note that there did not appear to be regional specificity for symptom domain. This is consistent with previous literature suggesting that “post-concussive” symptoms are not specific to mild TBI (Lagarde et al., 2014; Santhanam et al., 2019) and that PTSD and mild TBI may share common pathophysiological mechanisms (Kaplan et al., 2018), underscoring the difficulty in symptom attribution. Mild TBI, particularly with LoC, may interact with the expression of symptoms of PTSD. Thus, comprehensive assessment and treatment of TBI, particularly in Veteran populations, should include assessment of PTSD.

There was also a strong positive relationship between FA and RSA and BRS difference scores (supine 30° minus standing 90°), in all tracts but the CAB, across the full sample. It is typical for RSA and BRS to increase in the supine position compared with standing (90°), reflecting greater parasympathetic control (Lellamo et al., 1996; Laude et al., 2004). In our sample, lower values of FA in a key fronto-limbic pathway (the UNC), were associated with an abnormal autonomic response (lower RSA and BRS in the supine position). Increased BRS, as a measure of autonomic functioning, is associated with dampening of the locus-coeruleus-noradrenergic pathway, as would be expected in a relaxed state (e.g., when lying down). Dysfunction in this pathway is critical in the neurophysiology of anxiety related disorders. The locus-coeruleus projects to the basolateral amygdala, an important structure in the expression of PTSD and is within the same circuit regulated by prefrontal cortical-limbic projections (Daviu et al., 2019). Our findings suggest that this autonomic dysfunction is associated with reduced FA, possibly reflecting the effects of chronic stress on the brain due to poor management of allostatic load within the autonomic system. While the exact mechanisms underlying the relationship between white matter changes and chronic stress remain unclear, animal models suggest that chronic stress may alter the expression of genes responsible for pre-programmed cell death in oligodendrocytes due to changing cellular energy demands (Antontseva et al., 2020). Longitudinal investigations of the interaction between autonomic function and variability in white matter after TBI are necessary to draw conclusions about potential directionality and identify possible treatment targets. Increased stress after TBI may exacerbate physiological effects of injury with increased blood brain barrier permeability, amplified autonomic response to stressors, and poor sleep which may prolong recovery or perhaps increase the likelihood of chronic symptom presentation. It has been previously demonstrated that indicators of reduced autonomic function prior to exposure to a criterion A event (e.g., combat environments) are predictive of development of PTSD (Minassian et al., 2015).

It is notable that the strongest relationships between FA and stress measures (i.e., both global symptom severity and RSA/BRS difference scores) were found in the ATR and UNC across the full sample. Connecting orbitofrontal cortex to anterior temporal lobes, the UNC is particularly vulnerable to shearing injuries in TBI (Seo et al., 2012), and has been identified as a potential treatment target for deep brain stimulation treatment of PTSD (Hamani et al., 2022). The ATR, part of the anterior limb of the internal capsule, connects dorsolateral prefrontal cortex to the thalamus. Reduced white matter integrity within the fronto-subcortical pathways (ATR and UNC) may impair the ability to regulate emotional and autonomic functions (disinhibition of the amygdala), leading to increased overall stress burden (Williamson et al., 2013). Furthermore, the association between FA in the ATR/UNC and stress measures was strengthened in individuals with LoC in TBI, suggests that severity of TBI (LoC, compared with AoC) has an effect, as postulated by Williamson et al., 2013. Our findings underscore the importance of these limbic pathways in understanding the mechanisms underlying possible interactions between PTSD and TBI. Addressing this interaction with differential approaches to treatment is an important area of future research.

There also appeared to be a dissociation between stress measures and FA in the CAB. Global symptom severity was positively associated with FA in the CAB (possibly related to the loss of crossing fibers) in the LoC group and negatively associated with FA in the group without LoC. In contrast, there was no relationship between the CAB and autonomic functioning across both groups. This may reflect the relatively smaller role that parahippocampal cingulate projections play in autonomic regulation, despite their importance in maintenance of anxiety-avoidance learning, although the differing contributions of subdivisions within the cingulum remain unclear (Jones et al., 2013; Bubb et al., 2018).

The findings in the FMinor, the major interhemispheric connection between the frontal lobes, are more difficult to interpret. While the relationship between FA in the FMinor was significant in the group with LoC across both stress measures, the direction of effects is in contrast to the pattern observed in the UNC and CAB; higher values of FA were associated with both higher global symptom severity and more positive RSA/BRS difference scores (i.e., higher RSA/BRS during the 30° position). This is counter intuitive. On the one hand, it makes sense that higher FA is associated with normalized autonomic responses between standing and supine positions. On the other hand, greater severity of global symptoms is not consistent with this interpretation. It is possible that these findings represent different portions of variance across tracts and stress measures, reflecting heterogeneity in the manifestation of anxiety symptoms and non-emotional elements of autonomic control. However, this may reflect reduced interhemispheric coordination of frontal-limbic and autonomic regions (Xavier et al., 2018), and further research is needed to fully explore this possibility.

There are several important limitations to consider. Our sample is predominantly male, reflecting the demographics of the Veteran population in combat roles during the conflicts experienced by our cohort. Therefore, our findings may have limited generalizability for other populations, particularly for women with co-occurring TBI and PTSD, as is commonly seen in the context of inter-personal violence. This population is understudied and represents an important target of future research. An additional limitation is in our method for categorizing participants in mTBI and PTSD diagnostic groups. While the PCL-M has demonstrated high levels of diagnostic sensitivity, we did not use the gold-standard CAPS interview to assess for PTSD symptomatology due to time constraints, instead using a custom interview to establish criterion A occurrence. Similarly, while the OSU-TBI Identification method has been shown to have strong validity and reliability, there are many challenges to accurately diagnosing TBI using retrospective self-report particularly in military samples (Davenport et al., 2016). This issue is also true in civilian contexts as many people who experience mild TBI do not seek treatment. Third, like most studies of TBI and PTSD in humans, we have limited ability to determine the relative contributions of pre-morbid vulnerability, injury-related, and trauma-related factors, due to the lack of pre-injury assessment. In particular, pre-morbid characteristics such as cardiovascular disease burden and lifetime substance use history have been shown to impact the characteristics of white matter and is an important source of inter-subject variability in this sample. Longitudinal investigations are necessary to disentangle multifactorial contributions, including possible interactions with cerebrovascular risk factors and substance use, as well as possible sources of resiliency (e.g., social support), which would be salient to the Veteran population. Fourth, because FA is observed on the tract level for specific tracts, there may be more granular variations in microstructure within tract that were not explored, including other metrics of variability such as mean diffusivity, that could explain some of the counter-intuitive relationships observed in this study. The use of a pre-existing dataset (and older DWI sequence) precludes our ability to examine this more closely. The possibility of reduced crossing fibers contributing to higher values of FA warrants additional investigation, and has been previously reported (Figley et al., 2022). Finally, sample size, though relatively large given the integration of neuroimaging, autonomic, and neurobehavioral symptoms, is limited and replication of these findings is necessary. Though, it should be noted that others have previously reported differences in ventral-limbic pathway integrity in mild TBI and PTSD (Santhanam et al., 2019). In particular, our sample size limits our ability to fully parse the differences between mTBI with LoC, ambiguous mTBI (mTBI with alterations of consciousness alone), and those without a history of head strikes. Future research further probing heterogeneity within mTBI is necessary.

In conclusion, although this study cannot make determinations regarding causality, our findings add to the growing body of literature suggesting that history of TBI may interact with the symptoms of PTSD, regardless of the type of symptom (e.g., PTSD-related or post-concussive). Furthermore, we found evidence to suggest autonomic dysfunction and variability in ventral-limbic white matter pathways play a role in this relationship. Future research should explore autonomic functioning within this population to identify possible targets for intervention, public health strategies, and increase our understanding of the underlying mechanisms.

## Data availability statement

The data analyzed in this study is subject to the following licenses/restrictions: Limited datasets are available upon request. Requests to access these datasets should be directed to john.williamson@ufl.edu.

## Ethics statement

The studies involving humans were approved by the University of Florida Institutional Review Board. The studies were conducted in accordance with the local legislation and institutional requirements. The participants provided their written informed consent to participate in this study.

## Author contributions

AW: Conceptualization, Formal analysis, Methodology, Supervision, Visualization, Writing – original draft, Writing – review & editing. SB: Data curation, Formal analysis, Writing – original draft, Writing – review & editing. LJ: Data curation, Formal analysis, Writing – original draft, Writing – review & editing. DL: Methodology, Writing – review & editing. GL: Data curation, Formal analysis, Methodology, Writing – review & editing. JW: Conceptualization, Data curation, Funding acquisition, Investigation, Methodology, Project administration, Resources, Supervision, Writing – review & editing.
