# Supplementary material for: Regional associations of white matter integrity and neurological, post-traumatic stress disorder and autonomic symptoms in Veterans with and without history of loss of consciousness in mild TBI
Source: Front Neuroimaging. 2024 Jan 10;2:1265001. doi: 10.3389/fnimg.2023.1265001 (PMC10806103; doi:10.3389/fnimg.2023.1265001)
Supplement: Supplementary file 2 [file Table_1.docx]

|  | LOC (mean)  (n = 34) | No LOC (mean) (n=43) | Wilcoxon Rank Sum Test | P-value |
| --- | --- | --- | --- | --- |
| PCLM Total | 49.4 | 31.7 | W = 294 | <0.0001 |
| PCLM Avoidance | 20.5 | 12.2 | W = 402 | <0.0001 |
| PCLM Hyperarousal | 17.6 | 11.8 | W = 362.5 | <0.0001 |
| PCLM Reexperiencing | 13.3 | 8.74 | W = 402 | <0.0001 |
| NSI Total | 31.9 | 14.2 | W = 306 | <0.0001 |
| NSI Affective | 11.4 | 5.33 | W = 338 | <0.0001 |
| NSI Cognitive | 6.65 | 3.63 | W = 421.5 | <0.001 |
| NSI Somatic | 8.41 | 3.09 | W = 302.5 | <0.0001 |
| NSI Vestibular | 2.97 | 0.93 | W = 358.5 | <0.0001 |
