# Supplementary material for: Regional associations of white matter integrity and neurological, post-traumatic stress disorder and autonomic symptoms in Veterans with and without history of loss of consciousness in mild TBI
Source: Front Neuroimaging. 2024 Jan 10;2:1265001. doi: 10.3389/fnimg.2023.1265001 (PMC10806103; doi:10.3389/fnimg.2023.1265001)
Supplement: Supplementary file 3 [file Table_2.docx]

|  | FA Average (Center) | |
| --- | --- | --- |
|  | M | SD |
| ATR | 0.41 | 0.06 |
| CAB | 0.33 | 0.07 |
| CCG | 0.52 | 0.08 |
| ILF | 0.45 | 0.06 |
| SLF-P | 0.42 | 0.06 |
| SLF-T | 0.41 | 0.06 |
| UNC | 0.39 | 0.06 |
| FMinor | 0.49 | 0.06 |
| FMajor | 0.56 | 0.07 |
