# Supplementary material for: Regional associations of white matter integrity and neurological, post-traumatic stress disorder and autonomic symptoms in Veterans with and without history of loss of consciousness in mild TBI
Source: Front Neuroimaging. 2024 Jan 10;2:1265001. doi: 10.3389/fnimg.2023.1265001 (PMC10806103; doi:10.3389/fnimg.2023.1265001)
Supplement: Supplementary file 4 [file Table_3.docx]

Total Sample (n=65) LOC (n=30) No LOC (n=35)

| Position | BRS | RSA | LF HRV | HP | BRS | RSA | LF HRV | HP | BRS | RSA | LF HRV | HP |
| --- | --- | --- | --- | --- | --- | --- | --- | --- | --- | --- | --- | --- |
| 90° (Time 1) | 0.013 (0.01) | 4.65 (1.15) | 6.43 (0.98) | 788.64 (122.83) | 0.013 (0.01) | 4.66 (1.50) | 6.47 (0.85) | 795.81 (127.10) | 0.013 (0.01) | 4.64 (1.25) | 6.40 (1.10) | 782.484 (120.58) |
| 60° (Time 1) | 0.013 (0.01) | 4.76 (1.16) | 6.33 (1.11) | 819. 72 (136.14) | 0.014 (0.01) | 4.64 (1.89) | 6.20  (1.19) | 832.52 (143.13) | 0.012 (0.006) | 4.85 (1.15) | 6.40 (1.06) | 808.75 (130.94) |
| 30° (Supine) | 0.015 (0.01) | 5.37 (1.36) | 5.97 (1.22) | 937.34 (164.46) | 0.015  (0.01) | 5.44 (1.45) | 5.95 (1.40) | 952.64 (156.56) | 0.16 (0.009) | 5.32 (1.29) | 5.99 (1.08) | 924.22 (172.12) |
| 60° (Time 2) | 0.013 (0.01) | 4.81 (1.14) | 6.54 (1.09) | 820.13 (129.83) | 0.013 (0.01) | 4.79 (1.10) | 6.45 (1.02) | 825.53 (130.74) | 0.013 (0.08) | 4.83 (1.19) | 6.62 (1.16) | 815. 50 (130.78) |
| 90° (Time 2) | 0.013 (0.01) | 4.83 (1.09) | 6.59 (1.02) | 780.53 (118.52) | 0.013 (0.01) | 4.66 (1.05) | 6.47 (0.85) | 795.81 (127.1) | 0.013 (0.01) | 4.64 (1.25) | 6.40 (1.10) | 782.50 (120.60) |

*Note.* All reported in Mean (SD). BRS = Baroreceptor sensitivity, HP = Heart Period, LF HR = Low frequency heart rate variability, LOC = Loss of consciousness, RSA = Respiratory sinus arrhythmia.
