# Supplementary material for: Regional associations of white matter integrity and neurological, post-traumatic stress disorder and autonomic symptoms in Veterans with and without history of loss of consciousness in mild TBI
Source: Front Neuroimaging. 2024 Jan 10;2:1265001. doi: 10.3389/fnimg.2023.1265001 (PMC10806103; doi:10.3389/fnimg.2023.1265001)
Supplement: Supplementary file 4 [file Data_Sheet_1.DOCX]

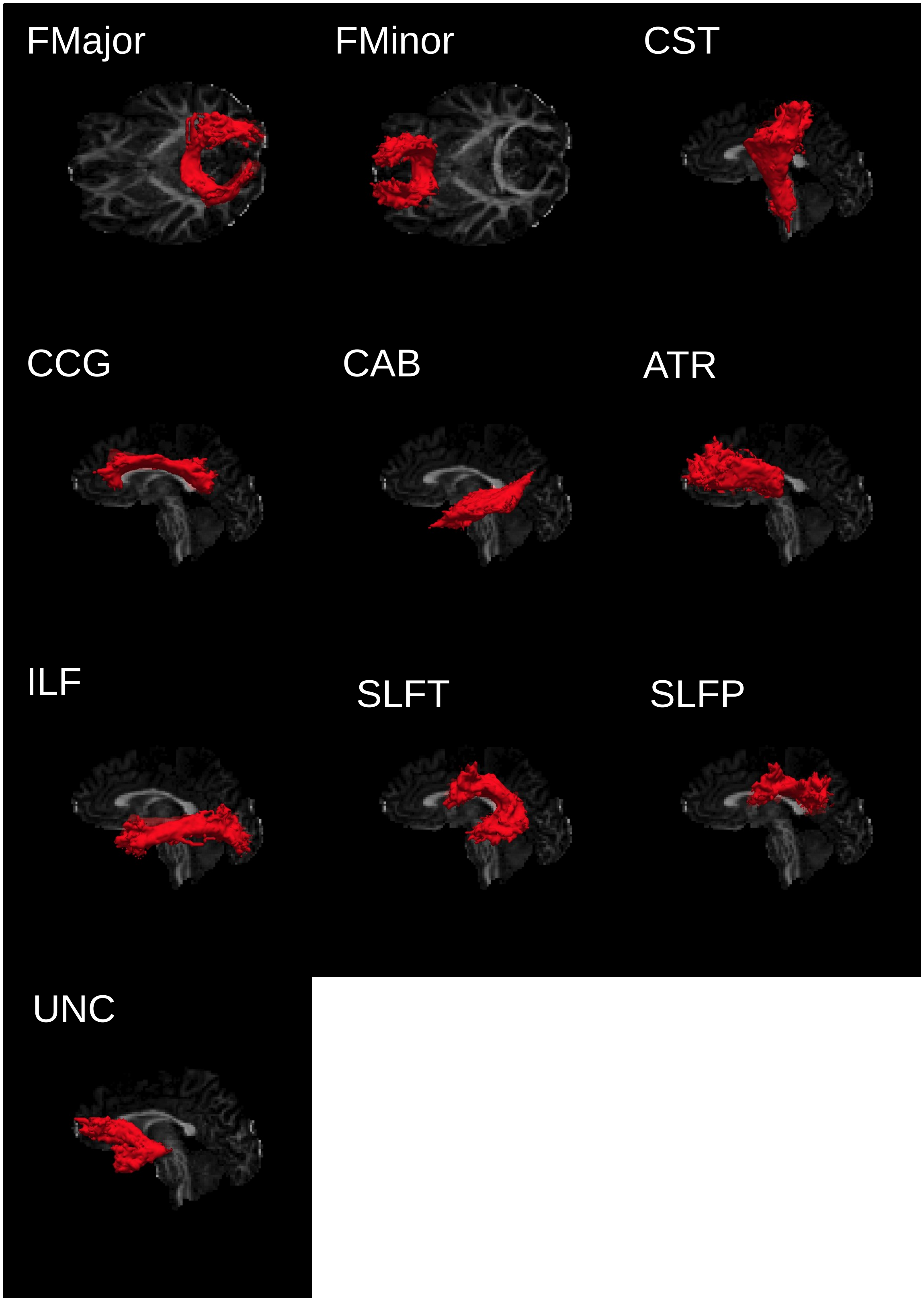
FMajor
FMinor
CST
CCG
CAB
ATR
ILF
SLFT
SLFP
UNC
